# Potential Effect of *SOX2* on the Cell Cycle of Wharton's Jelly Stem Cells (WJSCs)

**DOI:** 10.1155/2019/5084689

**Published:** 2019-06-02

**Authors:** Małgorzata Świstowska, Paulina Gil-Kulik, Arkadiusz Krzyżanowski, Tomasz Bielecki, Marcin Czop, Anna Kwaśniewska, Janusz Kocki

**Affiliations:** ^1^Department of Clinical Genetics, Medical University in Lublin, 20-080, Poland; ^2^Chair and Department of Obstetrics and Pathology of Pregnancy, Medical University of Lublin, 20-081, Poland; ^3^Department of Didactics and Medical Simulation, Medical University of Lublin, 20-093, Poland

## Abstract

The connective tissue of the umbilical cord contains stem cells called Wharton's jelly cells. These cells express core transcription factors (*NANOG*, *OCT4*, and *SOX2*). The protein product of the *SOX2* gene controls the cell cycle by interacting with cyclin D (directly and indirectly) and cycle inhibitors—p21 and p27, as well as two E2f3 protein isoforms. The aim of the study was to analyze the effect of *SOX2* on the cell cycle of stem cells of Wharton's jelly. The material for the study was the stem cells of Wharton's jelly isolated from 20 umbilical cords collected during childbirth. The stem cells collected were subjected to cytometric analysis, cell culture, and RNA isolation. cDNA was the starting material for the analysis of gene expression: *SOX2*, *CCND1*, *CDK4*, and *CDKN1B*. The studies indicate a high proliferative potential of the Wharton's jelly stem cells and the inhibitory effect of *SOX2* on the expression of the *CCND1* and *CDK4* gene.

## 1. Introduction

Wharton's jelly that forms umbilical cord plays an important role in ensuring vascular patency [1]. Stem cells are obtained from gelatinous connective tissue, subendothelium of umbilical vein, and umbilical cord blood. In the gelatinous connective tissue, rich in mucopolysaccharides and proteoglycans, there are umbilical cord matrix cells called the Wharton's jelly cells (WJCs) [2].

Phenotypically, umbilical cord cells present a number of antigens characteristic of mesenchymal stem cells found in adult human tissues, including CD44, CD73, CD90, and CD105 antigens. They do not express the common leukocyte antigen and CD14, CD31, CD56, and HLA-DR antigens [3–5], synthesize HLA-G, and have a higher proliferative potential and longer telomeres than the mesenchymal stem cells present in the tissues of the adult body [6–8].

WJCs express *NANOG*, *OCT4*, and *SOX2* core transcription factors, a gene characteristic of embryonic cells, *SSEA4*, *TRA-1-60*, and telomerase reverse transcriptase activity, suggesting their original, undifferentiated character [9, 10]. The core transcription factors, called nuclear transcription factors, are responsible for maintaining the state of pluripotency, self-renewal, and inhibition of stem cell differentiation.

Discovered and described in 1994 by Stevanovic et al., *SOX2* gene (SRY-Related HMG-Box Gene 2) is located in the long arm of chromosome 3, in the region 3q26.3-27 [11]. It belongs to the *SOX* gene family composed of 20 different genes divided into 8 groups (A, B, C, D, E, F, G, and H). The *SOX2* gene encodes the SOX2 protein composed of 317 amino acids [12]. The SOX2 protein, similar to other proteins encoded by *SOX* genes, has the HMG (High Mobility Group) domain built of approximately 80 amino acids [13]. Through the HMG domain, SOX proteins bind to the ATTGTT motif in DNA [14, 15].

The level of SOX2 protein expression depends on the cell type and degree of differentiation. The function of this protein in the cell is strictly dependent on its concentration, which is regulated on many levels, including transcription, posttranscription, and posttranslational levels [16].

The mechanism of action of SOX2 protein is based on interaction with other proteins leading to the formation of an active complex. Active complex controls many processes occurring in cells [16]. The SOX2 protein interacts with the NANOG protein, OCT4 protein, other proteins (ESRRB, KLF4, SALL1 and SALL4) that are transcription factors responsible for maintaining the self-resilience, and proteins responsible for chromatin remodeling (NuRD, Swi/Snf), DNA replication, and DNA repair [17–23]. SOX2 could also form an inhibitory complex. During mesendoderm development, MSX2 form an inhibitory complex with SOX2 by binding to the *SOX2* promoter [24].

The protein product of the *SOX2* gene controls the cell cycle by interacting with cyclin D (directly and indirectly) [25, 26]. In the scientific literature, there are also reports on the regulation of *SOX2* gene expression through proteins that inhibit the cell cycle—p21 protein [27] and p27 Kip1 [28], as well as two isoforms of E2f3 protein regulating the cell cycle as a result of interaction with the Rb protein [29].

## 2. Material and Methods

Stem cells were isolated from Wharton's jelly umbilical cord obtained during delivery from 20 patients of the Obstetrics Clinic and Pregnancy Pathology. The tests were carried out in accordance with the protocol and after obtaining the consent of the Bioethical Commission at the Medical University of Lublin (no. KE-0254/128/2014).

Stem cell isolation was performed using enzymatic digestion. A fresh part of the umbilical cord (5 cm) was rinsed in a phosphate-buffered saline (PBS) solution (Biomed, Lublin, Poland) with an antibiotic—0.5% solution of penicillin with streptomycin (PAA, Austria) and 0.5% amphotericin solution (PAA, Austria)—and then was cut into 2 mm diameter pieces of Wharton's jelly. Afterwards, the cord was digested in a collagenase solution (Sigma, USA) in 10 mg/30 ml of PBS at 37°C. The digested umbilical cord was passed through a 100 *μ*m diameter filter and centrifuged (10 minutes, room temperature, 800 RPM (rotations per minute)). Supernatant was removed, and then a 20% FBS solution (Gibco, USA) in PBS was added to neutralize the collagenase effect. The resulting mixture was centrifuged, the supernatant was removed, and the remaining pellet was suspended in 20 ml of the culture medium containing 10% FBS, 0.5% solution of penicillin with streptomycin, 0.5% amphotericin solution, and DMEM (Dulbecco's Modified Eagle Medium) (Gibco, USA) and then was placed in a TC Flask T25, Cell+ (Sarstedt, Germany) vessel intended for adherent culture.

The stem cell cultures of Wharton's jelly were incubated for 10 and 14 days at 37°C, in a 5% CO_2_ atmosphere with limited oxygen supply (not exceeding 4%). The culture medium was changed every 3 days.

Having cultured the adherent stem cells, the culture medium was removed and the cells were washed twice by heating to 37°C PBS-antibiotic solution. Medium remains were removed. Next, 1 ml of a warm solution of PBS was added to the washed cells, and cell scraper (Sarstedt, Germany) was applied to detach the cells from the walls of the vessel. Cells suspended in PBS buffer were divided into 2 aliquots of 0.5 ml, transferred to Eppendorf tubes, and centrifuged (10 minutes, room temperature, 800 RPM). The supernatant from the pellet was removed. The cell pellet was subjected to further procedures.

The cytometric analysis of stem cells of Wharton's jelly for the CD34+/CD90+/CD105+ phenotype was performed according to the procedure in the publication “Phenotypic Characterization of Adherent Cells Population CD34+ CD90+ CD105+ Derived from Wharton's Jelly” [30]. Cytometric analysis was done for 10 samples. Each sample was analyzed once. The cytometric analysis was carried out in a MoFlo XDP cell sorter (Beckman Coulter) using the Summit software and Kaluza software and FlowSight cytometer (Amnis) using the Amnis software.

Cell proliferation analysis was performed using the Cell Trace CSFE Cell Proliferation Kit (Invitrogen, USA). After 10 days of cell culture, CellTrace loading solution containing CFSE (carboxyfluorescein diacetate succinimidyl ester) was added to cell cultures according to the attached procedure for adherent cells. The cytometric analysis of cell proliferation was done four days later using the FlowSight cytometer (Amnis, USA) and Amnis software.

Total RNA was isolated using the modified method of Chomczyński and Sacchi. Suspended in 500 *μ*l PBS, the stem cells were centrifuged (10 minutes, room temperature, 800 RPM); the supernatant was removed. RNA was obtained from the obtained cell pellet using the TRI reagent (Sigma, USA), chloroform (POCH, Poland), and isopropanol (Sigma, USA). Next, after measuring the concentration of the acid obtained, the RNA reverse transcription reaction was performed by means of a commercially available kit (High-Capacity Reverse Transcription Kit cDNA, Applied Biosystems, USA) and according to the attached procedure.

The study of *SOX2*, *CCND1*, *CDK4*, and *CDKN1B* expression was performed using the real-time PCR method. cDNA, probes: *SOX2* (Hs0153049_s1, Applied Biosystems, USA), *CCND1* (Hs00765553_m1, Applied Biosystems, USA), *CDK4* (Hs00262861_m1, Applied Biosystems, USA), and *CDKN1B* (Hs00153277_m1, Applied Biosystems, USA) and Master Mix buffer (Applied Biosystems, USA) were used for the analysis. The real-time PCR reaction, after the initial 10-minute denaturation at 95°C, was carried out according to the following scheme—40 cycles: 15 seconds at 95°C and 60 seconds at 60°C. Each sample was tested in duplicate. The reaction was carried out in the StepOnePlus Real-Time PCR System.

Gene expression analysis was performed using the StepOne Software v.2.2.2 and Expression Suite Software v.1.0.3.165 from Applied Biosystems.

For further calculations, the mean value ΔCt of individual samples normalized to endogenous control—*GAPDH* (Hs99999905_m1, Applied Biosystems, USA)—was used. To determine the relative gene expression (RQ), the following formula was used: RQ = 2^–ΔΔCT^ [31].

Statistical analysis was subjected to the final result which was the logRQ value of each gene expression. The statistical analysis was performed in the Statistica12 software using the Kruskal-Wallis ANOVA test and the multiple comparison test (*n* = 20, where *n* represents the number of patients from whom Wharton's jelly stem cells were obtained). Three levels of significance were determined: *p* < 0.05, *p* < 0.01, and *p* < 0.001.

**Figure 1:**
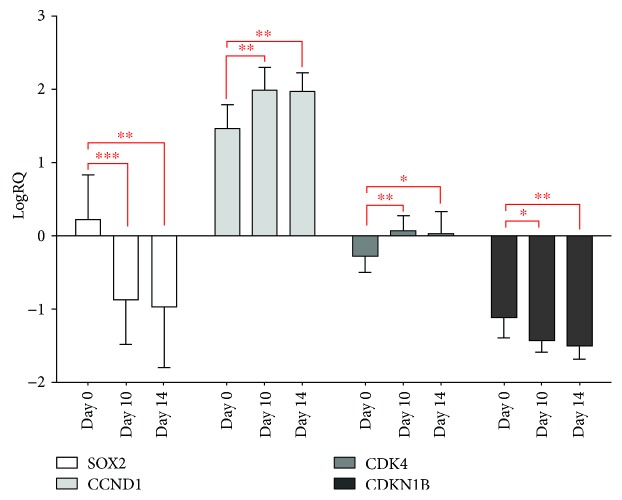
Graph showing the mean and standard deviation for logRQ of the *SOX2*, *CCND1*, *CDK4*, and *CDKN1B* gene in Wharton's jelly stem cells (nonbred: day “0” and after 10 and 14 days of culture). ^∗^*p* < 0.05, ^∗∗^*p* < 0.01, and ^∗∗∗^*p* < 0.001.

All of the statistical details of the experiments can be found in Results and in Figure 1.

## 3. Results

**Figure 2:**
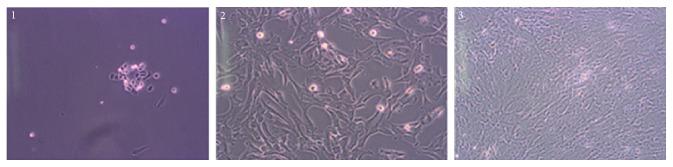
Wharton's jelly stem cells (1: on day 4 of culture; 2: 10 days of culture; 3: 14 days of culture). Microscopic image at 200x magnification (10x eyepiece, 20x objective). Own photo taken using the Olympus CKX41 inverted microscope and the Olympus XC50 camera.

As a result of the in vitro cultivation, the adherent properties of Wharton's jelly stem cells to the walls of culture vessel were confirmed. In the process of culturing, the cells presented proliferation capacity forming a monolayer around day 10 of culture. They formed colonies morphologically resembling fibroblasts. Observation results (microscopic images) are shown in Figure 2.

**Figure 3:**
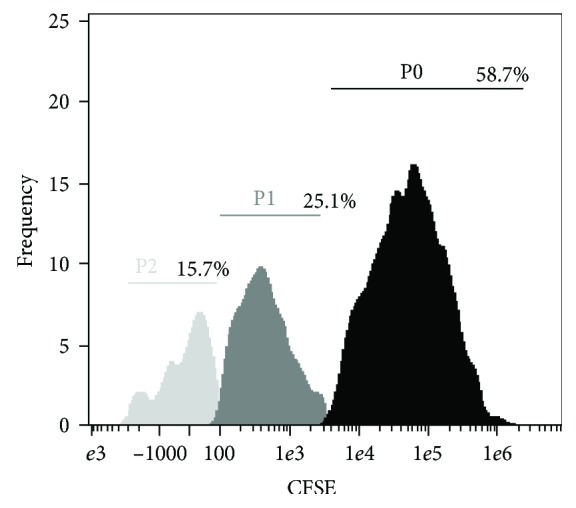
Histogram with 3 generations of Wharton's jelly stem cells. P0: population 0; P1: population 1; P2: population 2.

To determine cell cycle progression, analysis of proliferation with the CellTrace CSFE Kit was done. Cytometric analysis showed proliferative potential of the examined cells.  Figure 3 shows histogram with 3 generations of stem cells of Wharton's jelly (emission peaks from CSFE dye bonded covalently to intracellular amines).

**Figure 4:**
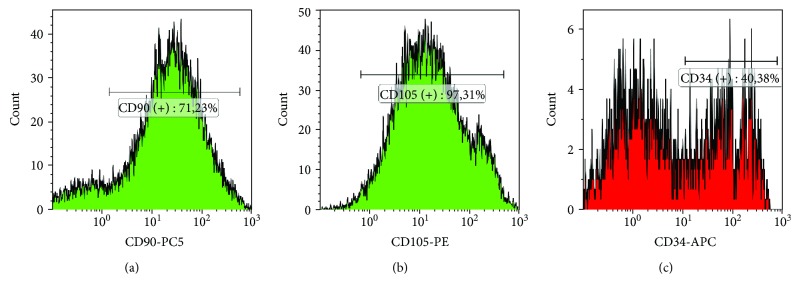
Histograms. Expression of CD34, CD90, and CD105 surface antigens in stem cells of Wharton's jelly. Own photos obtained from cytometric analysis carried out on a MoFlo XDP cell sorter (Beckman Coulter), generated using the Kaluza software.

To determine the phenotype of the isolated stem cells of Wharton's jelly, cytometric analysis was performed using the MoFlo XDP cell sorter (Beckman Coulter) and the FlowSight flow cytometer (Amnis).  Figure 4 presents exemplary histograms depicting fluorescence intensity of surface antigens.

**Figure 5:**
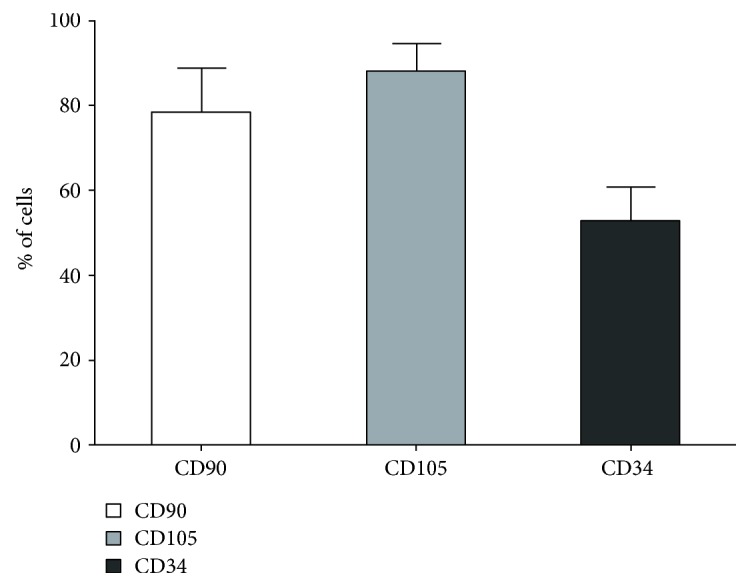
Mean percentage of stem cells of Wharton's jelly expressing the surface antigens tested during cytometric analysis.

Expression of CD105 antigen exhibited 88.04% WJSC and expression of CD90 antigen 78.29% WJSC, whereas expression of CD34 antigen 52.35% WJSC (Figure 5).

**Figure 6:**
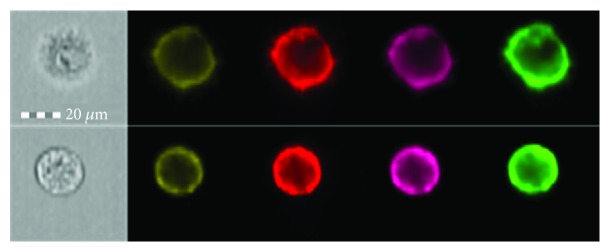
Wharton's jelly stem cells expressing CD34, CD90, and CD105 antigens. Cell morphology and fluorescence in individual channels of the FlowSight cytometer (Amnis). From the left: gray: cell morphology; yellow: CD105-PE antigen; red: CD90-PC5 antigen; pink: SSC (side scatter); green: CD34-APC antigen.

The pictures in Figure 6 show the morphology and fluorescence of the exemplary stem cells of Wharton's jelly in individual channels. The image was obtained during the cytometric analysis on the FlowSight apparatus (Amnis).

In our studies, the expression of the following genes was demonstrated: *SOX2*, *CCND1*, *CDK4*, and *CDKN1B* in both noncultured cells and cells subjected to cell culture.

Statistically significant differences (*p* < 0.05) were determined by the Kruskal-Wallis ANOVA test for the logRQ values of all the tested genes between the nongrown stem cells of Wharton's jelly and stem cells of the Wharton's jelly after 10 and 14 days of culture (Figure 1).

In order to accurately identify statistically significant differences, the analysis was extended by a multiple comparison test. The multiple comparison test showed statistically significant differences (*p* < 0.05) for the logRQ values of the *SOX2*, *CCND1*, *CDK4*, and *CDKN1B* genes between the Wharton's jelly stem cells, nonbred and after 10 days of culture, as well as noncultured and after 14 days of culture. There were no statistically significant differences for the logRQ values of particular genes between the Wharton's jelly stem cells after 10 and 14 days of culture (Figure 1).

In our studies, a significant decrease in the level of *SOX2* gene expression in stem cells of jelly substance was observed as a result of cell culture compared to the level of expression prior to cultivation (*p* < 0.05) (Figure 1).

The highest level of *SOX2* gene expression was observed in nongrown stem cells (the mean for logRQ of the *SOX2* gene is 0.23), while the lowest level of *SOX2* gene expression was recorded in stem cells after 14 days of culture (the mean for logRQ of the *SOX2* gene is -0.97) (Figure 1).

During cell culture, there was a significant increase in the expression of the *CCND1* gene and *CDK4*. Stem cells of Wharton's jelly after the first day of cultivation exhibited the highest expression level of the *CCND1* gene (the mean for logRQ of the *CCND1* gene is 1.99), while the lowest level of *CCND1* gene expression was observed in noncultured stem cells (the mean for logRQ of the *CCND1* gene is 1.47) (Figure 1).

The highest level of *CDK4* gene expression was observed in Wharton's jelly stem cells after day 10 of culture (the mean for logRQ of the *CDK4* gene is 0.07). The lowest expression level of the *CDK4* gene was displayed by Wharton's jelly stem cells prior to culture (the mean for logRQ of the *CDK4* gene is -0.27) (Figure 1).

In addition, the effect of changes in *SOX2* gene expression on the expression of *CCND1* and *CDK4 kinase* was analyzed. The Wharton's jelly stem cells did not show a gene expression. Moreover, the expression of the *CCND1* gene and *CDK4 kinase* significantly increased with *SOX2* gene decrease (*p* > 0.05) indicating the inhibitory effect of *SOX2* on the expression of *cyclin D1* and *CDK4 kinase* (Figure 1).

In addition, there was a decrease in *CDKN1B* gene expression during cell culture. The decrease in *CDKN1B* gene expression was accompanied by a decrease in *SOX2* gene expression.

In stem cells of Wharton's jelly, the highest level of *CDKN1B* gene expression was observed prior to culture (the mean for logRQ of the *CDKN1B* gene is -1.11). A similar level of expression of the *CDKN1B* gene was demonstrated by stem cells after day 10 of culture (the mean for logRQ of the *CDKN1B* gene is -1.42) and stem cells after day 14 of culture (the mean for logRQ of the *CDKN1B* gene is -1.50) (Figure 1).

## 4. Discussion

In the light of recent studies, the *SOX2* gene can also regulate the cell cycle, migration, and cell adhesion [32–34] in addition to its ability to self-renew and differentiate. Depending on the type of stem cell and concentration, the SOX2 protein can activate [32–34] or inhibit the expression of the *CCND1* gene [26].

In our own studies, a significant increase in the expression of the *CCND1* gene and *CDK4 kinase*, as well as the decrease in *SOX2* gene expression in the Wharton's jelly stem cells, was observed during cell culture.

Research on the effect of *SOX2* gene expression on the progression of the cell cycle and the ability to differentiate was conducted by Han et al. [32] and Yoon et al. [34]. Yoon et al. observed that in cultures with low cell density, the increase in the expression of pluripotency markers is accompanied by an increase in cell proliferation, the percentage of cells in the S and G2/M phases of the cell cycle, the expression of cyclins A, B, and D, as well as kinases, CDK2 and CDK4, and the ability to chondrogenesis and adipogenesis. To confirm their theory of linking the expression of pluripotency genes to cell proliferation and differentiation, scientists introduced intervening siRNAs to silence the expression of the *SOX2* gene. As a result of the experiment, there was a decrease in SOX2 expression at the protein level, which was accompanied by a decrease in proliferation and ability to differentiate. The obtained results indicated that the SOX2 protein plays a role in maintaining the proliferation and multiplication of MSC cells [34].

Transfection with the *OCT4-IRES-SOX2* plasmid vector of mesenchymal stem cells by Han et al. reduced the percentage of cells in the G1 cycle phase and the increase in the number of cells in the S phase indicating proliferation. In addition, analysis at the protein level using Western blot produced an increase in the content of cyclin D1 in cells, which may indicate the transition from the G1 phase to the S phase. Infected cells were also characterized by an increase in differentiation potential to adipocytes and osteoblasts as evidenced by the higher accumulation of adipocyte dyes (oil red O) and osteocytes (azarin S) as well as an increase in the expression of genes characteristic of fat cells—PPARgamma, lipoprotein lipase, and bone tissue—collagen I, and osteocalcin. The overexpression of the *SOX2* gene led to the growth of markers characteristic for mesoderms, neuroderms, and trophopodectomies [32].

Liu et al. using retroviral transfection introduced the *SOX2* gene to isolate from the dental pulp of the stem cells and then studied the effect of overexpression on the ability to proliferate, migration, and adhesion of infected cells. Cytometric analysis of the cell cycle showed that a greater percentage of cells overexpressing the *SOX2* gene were in the S phase of the cycle and a smaller one in the G1/G0 phase as compared to the control cells. In addition, increased expression of the *SOX2* gene resulted in an increase in the proliferation index, FBS-induced migration, and the ability to adhere to cells induced with fibronectin. By using RNA microarray technology, researchers have proven that *SOX2* can regulate cell adhesion and cell division. As a result of the reaction, the qPCR showed an increase in the expression of the cell cycle genes (*cyclins A1*, *D1*, and *E* and *CDK2 kinase*), which are responsible for migration (*PI3K* and *EDN1*) and cell adhesion (*CLDN1*, *CLDN2*, *JAM3*, *HRAS*, and *F11L*) in cells from the gene *SOX2*. Furthermore, the researchers noted an increase in the amount of SOX2 protein in infected cells [33].

Greco et al., using siRNA, silenced the expression of the *OCT4* gene, whose protein product forms a heterodimeric complex with the SOX2 protein. As a result of the experiment, there was not only a decrease in the expression of *SOX9*, *HDACI*, and *PH-4* genes, responsible for differentiation towards mesoderm, but also a decrease in genes related to the cell cycle (*cyclins A1*, *B1*, and *D1*, *CDK2 kinase*, and *CDK4*) as well as increased expression of the *p21* inhibitor resulting in cell output from the cycle [35].

Similar observations were made by Riekstina et al. The decrease in *OCT4* gene expression in bone marrow stem cells led to the inhibition of divisions, exit from the cycle, and passage of cells to the resting phase [36].

Regulation of *CCND1* gene expression by SOX2 protein takes place both directly and indirectly [25, 26]. Direct regulation is based on the attachment of SOX2 protein to binding sites of the *CCND1* gene promoter [26].

Hagey and Muhr, as a result of research conducted on neural stem cells, identified in the region of the gene promoter *CCND1* 9 sites binding protein SOX2 characterized by a greater or lesser affinity to this protein. In addition, they found that the interaction of the SOX2 protein with the promoter of the *CCND1* gene inhibits its expression. Further research into the effect of attaching SOX2 protein to specific sites in the *CCND1* gene promoter region has yielded a surprising result. The mutation of sites showing greater affinity for the SOX2 protein only contributed to a slight increase in the expression of *CCND1* as opposed to the mutation of low affinity sites, which resulted in a significant increase in the expression of this gene. In addition, the researchers analyzed the effect of interaction of SOX2 protein with betacatenin, Lef/Tcf proteins, and their Gro/Tle protein cofactors on the expression of cyclin D1. The interaction of SOX2 protein with betacatenin at binding sites with higher affinity abolished the activating nature of betakatein for *CCND1* expression. Attachment of Lef1 protein enhances SOX2 inhibitory properties, and Tcf7L1 inhibits *CCND1* expression only in the presence of SOX2 protein. The Tle protein induces a synergistic effect on the expression of CCND1. The increase in *SOX2* gene expression enhances the interaction between Lef1 and Tle1 as a result of the interaction of SOX2 protein with the Tle by the C-terminal, non-DNA-binding domain [26].

Card et al. in their studies on the effect of *OCT4*, *SOX2*, and *NANOG* gene expression on the cell cycle in embryonic stem cells observed that the expression of *miR-302a* is dependent on *SOX2* and *OCT4*. In addition, the protein products of these genes attach to the binding sites of the *miR-302* cluster promoter, one of those whose target is cyclin D. As a result of miR-302 activation, the concentration of cyclin D increases, accompanied by an increase in the number of cells in the S phase and decrease in the G1 phase, which indicates the indirect participation of *SOX2* and *OCT4* in the regulation of the cell cycle [25].

The expression of the *SOX2* gene can be regulated by cell cycle inhibitors, e.g., p27 kip. Li et al. during the differentiation of pluripotent stem cells, using retinoic acid, noted a decrease in *SOX2* gene expression with the increase of p27 Kip1 protein expression [28].

In contrast to the experiment conducted by Li et al., in our own studies, due to stem cell culture of Wharton's jelly, the decrease in *SOX2* gene expression was accompanied by a decrease in *CDKN1B* gene expression, which may indicate a different regulation of *SOX2* gene expression by *CDKN1B* in the examined cells.

## 5. Conclusion

In conclusion, it was noted that in the process of cell culture, a significant decrease in *CDKN1B* gene expression and an increase in the expression of the *CCND1* gene and *CDK4 kinase* were observed, which may indicate a high proliferative potential of cells derived from the Wharton's jelly of the umbilical cord.

In addition, in the examined umbilical cord parent cells, an increase in the expression of the *CCND1* gene and *CDK4* gene and a decrease in *CDKN1B* gene expression (*p* < 0.05) were accompanied by a decrease in SOX2 gene expression indicating potential inhibitory effects of *SOX2* on the expression of *cyclin D1* and *CDK4 kinase*.

The research conducted on the stem cells of the Wharton's jelly umbilical cord sheds new light on the current reports. In addition, they confirm the potential inhibitory effect of SOX2 protein on the expression of *cyclin D1*. This may indicate a similar mechanism of mutual regulation of *SOX2* gene expression and expression of cell cycle genes in stem cells of Wharton's jelly and parental nerve cells.

## Data Availability

The data used to support the findings of this study are included within the article and can be available from the corresponding author.
